# A Red Herring in the Cerebrospinal Fluid: Xanthochromia Due to Mixed Etiology in the Absence of Subarachnoid Hemorrhage

**DOI:** 10.7759/cureus.83705

**Published:** 2025-05-08

**Authors:** Neville Shery, Rafid Mustafa, Mohammed Al-Hamas

**Affiliations:** 1 General Medicine, Pilgrim Hospital, Boston, GBR; 2 Acute Medicine, Pilgrim Hospital, Boston, GBR; 3 Acute Internal Medicine, Pilgrim Hospital, Boston, GBR

**Keywords:** acute subarachnoid haemorrhage, cerebrospinal fluid (csf), headache medicine, varicella encephalitis, xanthochromia

## Abstract

Xanthochromia in cerebrospinal fluid (CSF) is most commonly associated with subarachnoid hemorrhage (SAH), but other etiologies must be considered when initial imaging is unremarkable. We present the case of a 50-year-old woman with a sudden, severe headache. Computed tomography (CT) of the head was normal, but lumbar puncture revealed xanthochromia, elevated CSF protein (139.5 mg/dL), and no red or white blood cells. Varicella zoster virus (VZV) was detected by CSF polymerase chain reaction (PCR), and MRI demonstrated chronic cervicomedullary stenosis without evidence of hemorrhage or encephalitis. The patient was treated empirically with intravenous acyclovir and fully recovered. The findings suggest a mixed etiology for xanthochromia involving partial mechanical obstruction of CSF flow and a subclinical viral process. This case highlights the importance of considering infectious, inflammatory, and structural causes in the differential diagnosis of xanthochromia when SAH is excluded.

## Introduction

Xanthochromia - the yellow discoloration of cerebrospinal fluid (CSF) - is most commonly associated with subarachnoid hemorrhage (SAH), where red blood cells enter the subarachnoid space and subsequently degrade into oxyhemoglobin and bilirubin [1]. In clinical practice, xanthochromia detected via lumbar puncture (LP) is a key diagnostic marker for SAH, particularly when initial computed tomography (CT) imaging is unremarkable. The most frequent cause of false-positive xanthochromia is a traumatic LP, where introduced red blood cells degrade in vitro [2].

However, xanthochromia is not specific to hemorrhage. Other recognised causes include elevated CSF protein, Froin’s syndrome, traumatic LP with delayed analysis, serum hyperbilirubinaemia, and bacterial meningitis [1,3-6]. Viral infections are rarely associated with xanthochromia but may contribute through mechanisms such as hemorrhagic necrosis or hyperproteinaemia, as described in a Japanese study of herpes simplex encephalitis [7]. In some cases, viral infections may also impair CSF flow and contribute to Froin’s syndrome [8].

We report a rare case of xanthochromia in a patient with chronic cervical spinal stenosis, marginally elevated CSF protein (139.5 mg/dL), and a positive CSF PCR for varicella zoster virus (VZV). In the absence of red blood cells and without markedly elevated protein, a single unifying cause could not be identified. This case highlights a possible mixed aetiology involving subclinical viral infection, partial mechanical obstruction, and chronic inflammatory disease. It underscores the importance of considering a broad differential diagnosis for xanthochromia when SAH is excluded.

## Case presentation

A 50-year-old woman presented to the emergency department with a sudden-onset, crushing headache, described as the worst pain of her life. There were no clear exacerbating or relieving factors. The headache was accompanied by blurred central vision but no photophobia, neck stiffness, balance disturbance, speech difficulties, or motor or sensory deficits. She had no history of recurrent headaches, and no rash was observed. Neurological examination revealed no focal abnormalities.

On admission, her vital signs were: blood pressure 108/75 mmHg, heart rate 76 beats per minute, temperature 36.9 °C, oxygen saturation 98% on room air, and respiratory rate 20 breaths per minute.

Her past medical history included significant cervical canal stenosis with cervicomedullary narrowing. She had undergone decompression surgery in 2020 and occipital-cervical fusion in 2022. Additional history included chronic obstructive pulmonary disease and a prior transient ischemic attack. There was no history of migraine, recent trauma, or other neurological disorders.

Her medications included clopidogrel, budesonide/formoterol, salbutamol, atorvastatin, duloxetine, and gabapentin.

An urgent non-contrast computed tomography (CT) head scan was performed to evaluate for subarachnoid hemorrhage (SAH), which revealed no acute abnormality.

Blood tests on admission showed an elevated white blood cell count, with normal C-reactive protein (CRP) (Table 1).

**Table 1:** Summary of blood test results on admission

Blood Test	Result	Range/Units
Sodium	143	133 – 146 mmol/L
Potassium	4.1	3.5 - 5.3 mmol/L
Urea	3.0	2.5-7.8 mmol/L
Creatinine	69	59-104 µmol/L
Hemoglobin	147	130-180 g/L
White blood cells	13.8	4.3-11.2x10^9^/L
Neutrophils	6.28	2.1-7.4x10^9^/L
Lymphocytes	1.62	1.0-4.0x10^9^/L
Monocytes	0.91	0.2-0.84x10^9^/L
Eosinophils	0.28	0.1-0.44x10^9^/L
Basophils	0.07	0.02-0.10 x10^9^/L
Bilirubin	8	0-21 µmol/L
Total protein	72	60-90 g/L
C-reactive protein	21	<5 mg/L

According to the National Institute for Health and Care Excellence (NICE) guidelines, if a CT head scan performed within 6 hours of symptom onset in a patient with suspected subarachnoid hemorrhage is negative, a lumbar puncture should be considered [4]. A non-traumatic lumbar puncture was performed 12 hours after symptom onset (results in Table 2).

**Table 2:** CSF results CSF: cerebrospinal fluid

CSF Test	Result	Reference Range
Colour	Clear, colourless	Clear, colourless
Red Blood Cells (×10¹²/L)	0	0
White Blood Cells (×10⁹/L)	0	0–5 ×10⁶/L (i.e., <5 cells/µL)
CSF protein (mg/dL)	139.5	15.0–45.0 mg/L
Xanthochromia	Positive (bilirubin detected)	Negative (no bilirubin or oxyhemoglobin)
Net Bilirubin Absorbance (476 nm)	0.008	<0.007 absorbance units
Oxyhemoglobin	<0.020	<0.020 absorbance units

The CSF protein level (139.5 mg/dL) was significantly elevated, suggesting impaired CSF circulation. The absence of red blood cells excluded traumatic LP as a source of bilirubin. A white cell count of zero would also be atypical for viral encephalitis. Net bilirubin absorbance above 0.007 confirmed the presence of xanthochromia.

The patient was initially started on tranexamic acid 1 g three times daily for suspected SAH. A CT angiogram of the cerebral vessels was subsequently performed and found to be normal, ruling out aneurysm. Neurosurgical consultation was sought for further evaluation.

CSF cultures and viral polymerase chain reaction (PCR) were performed, with varicella zoster virus (VZV) detected in the sample.

The patient was treated empirically for suspected central nervous system (CNS) infection with intravenous acyclovir for 7 days, along with supportive care including IV fluids and analgesia.

**Figure 1:**
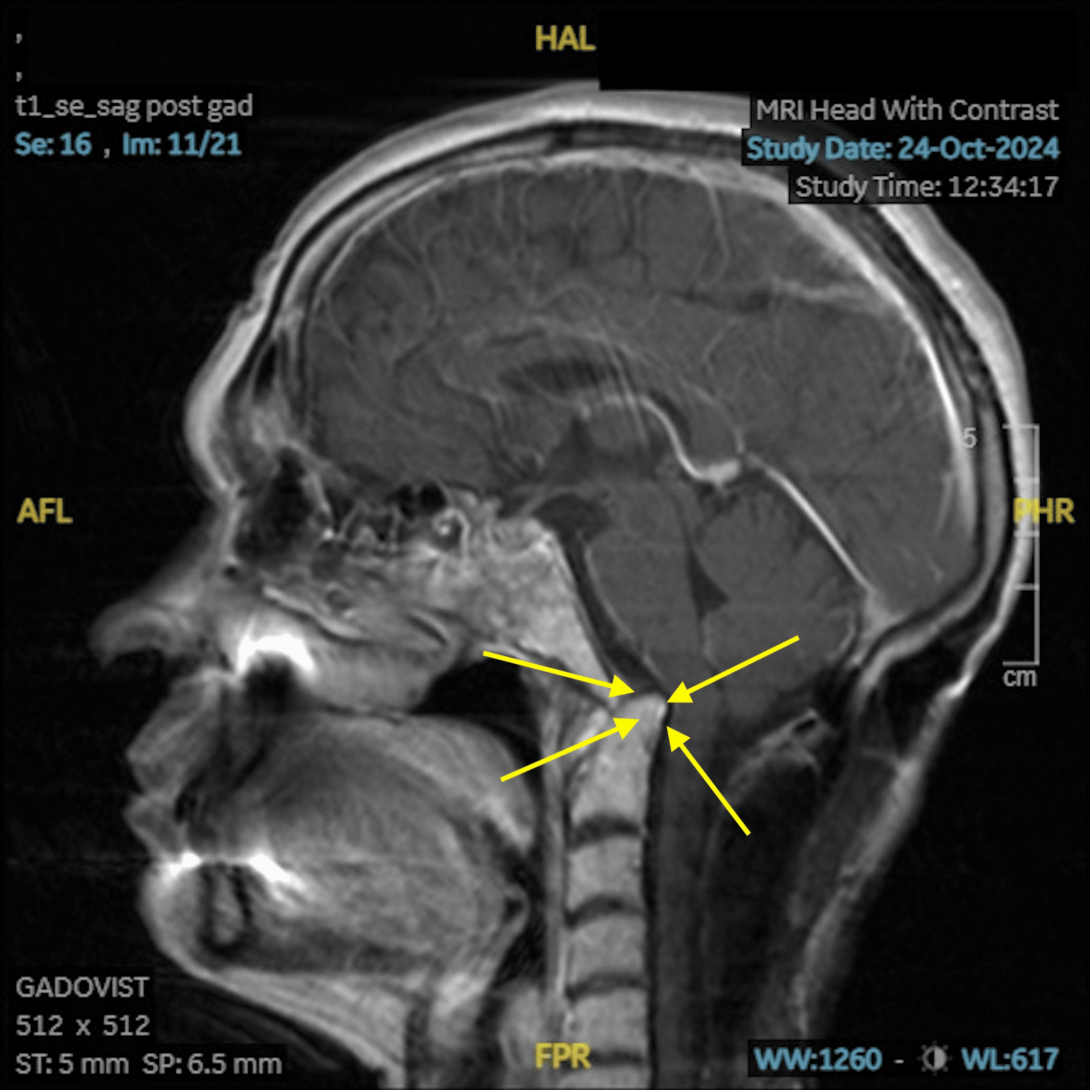
Sagittal T1-weighted post-contrast MRI Sagittal T1-weighted post-contrast MRI of the brain and upper cervical spine showing basilar invagination, with superior displacement of the odontoid process into the foramen magnum, resulting in cervicomedullary junction narrowing.

**Figure 2:**
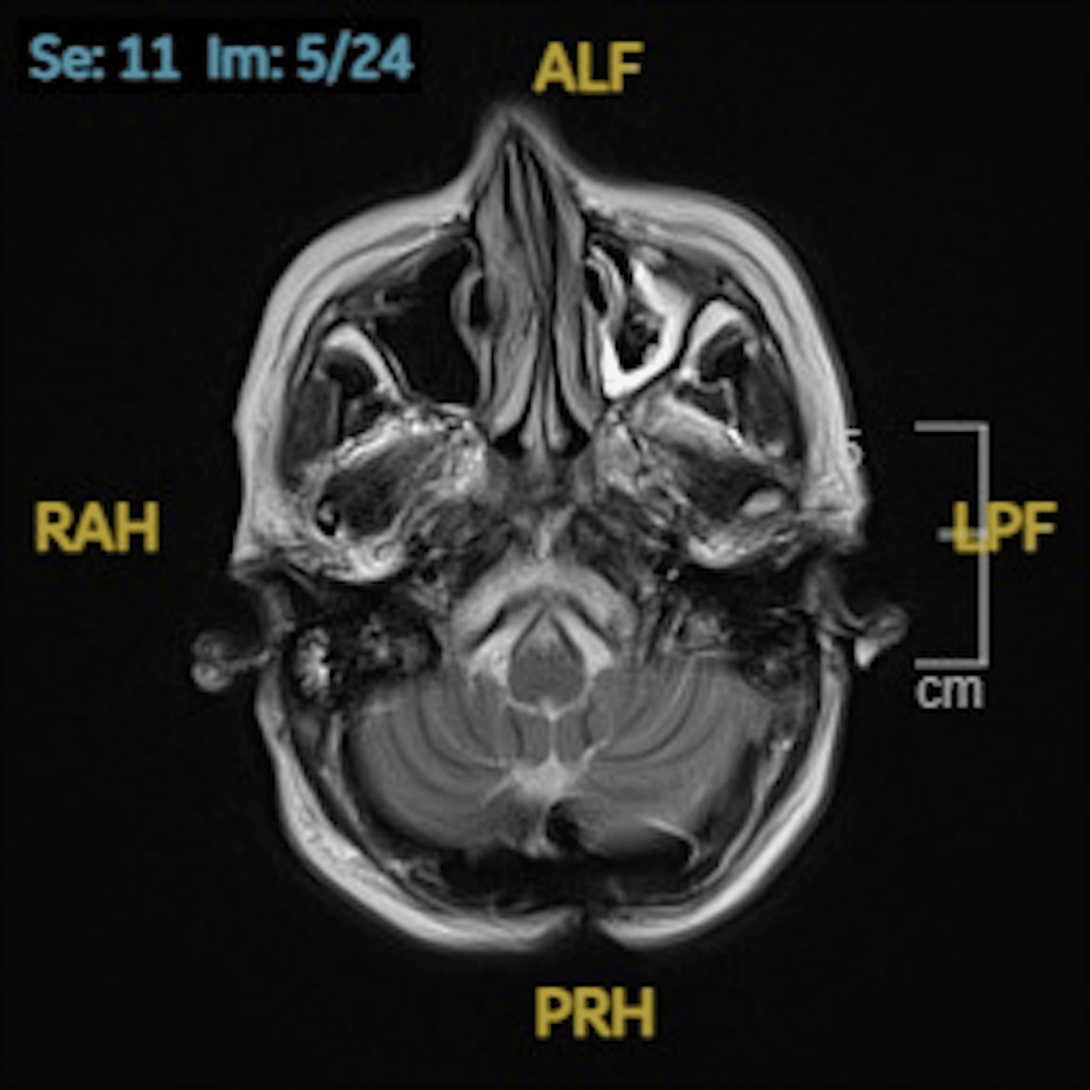
Axial T2-weighted MRI Brain Axial T2-weighted MRI of the brain at the cervicomedullary junction. There is a mild reduction of cerebrospinal fluid in the anterior compartment compared to the posterior compartment. There is no evidence of brain stem compression.

An MRI of the brain on day 5 showed no acute intracranial pathology or radiological features of encephalitis. However, it confirmed chronic basilar invagination at the foramen magnum with cervicomedullary junction stenosis-unchanged from prior imaging in 2022 (Figures 1, 2).

The patient’s headache gradually resolved over the 7-day period and fully subsided following completion of intravenous acyclovir. The transient nature of her symptoms, combined with a positive VZV PCR and the absence of acute radiological abnormalities, suggests a subclinical central nervous system (CNS) infection as the likely cause, with underlying cervicomedullary compression potentially predisposing to impaired cerebrospinal fluid dynamics.

## Discussion

Xanthochromia, derived from the Greek term meaning “blond colour,” refers to yellow pigmentation of the cerebrospinal fluid (CSF). It is classically considered a hallmark of subarachnoid hemorrhage (SAH), but alternative causes must be considered in the appropriate clinical context [1]. Xanthochromia occurs when red blood cells in the CSF lyse and are metabolised from oxyhemoglobin (pink) to bilirubin (yellow). Spectrophotometric analysis for the presence of oxyhemoglobin and bilirubin is the gold standard for detecting xanthochromia and is superior to visual inspection alone [1].

In suspected SAH, an urgent non-contrast CT head scan should be performed, as per NICE guidelines [9]. Beyond the initial diagnostic window, sensitivity declines, and lumbar puncture (LP) is recommended if clinical suspicion remains high. In this case, an LP was performed despite negative CT findings. Perry et al. demonstrated that CT followed by LP is sufficient to exclude SAH; however, they also noted that xanthochromia has high sensitivity but poor specificity, with all false positives in their study attributed to traumatic LPs (RBC > 5 × 10⁶/L) [2].

Several documented non-hemorrhagic causes of xanthochromia include: increased CSF protein (≥150 mg/dL) due to enhanced bilirubin binding, bacterial meningitis, Froin’s syndrome, traumatic LP with delayed analysis, serum hyperbilirubinaemia, and serum hyperproteinaemia (>10-15 g/dL) [1, 4, 5]. In this patient, serum bilirubin and total serum protein were within normal limits. Traumatic LP was excluded based on the absence of red blood cells in the CSF [2]. Froin’s syndrome was considered unlikely, as it typically requires markedly elevated CSF protein levels (>500 mg/dL) and spontaneous coagulation of the CSF.

Viral encephalitis typically presents with fever, altered mental state, and CSF pleocytosis [10]. In this case, CSF PCR for varicella zoster virus (VZV) was positive, raising the possibility of a low-grade or subclinical central nervous system (CNS) infection, despite the absence of classical clinical or radiological signs. Subclinical VZV reactivation has been reported in immunocompetent individuals [11]. Furthermore, a Japanese study of 108 patients with herpes simplex encephalitis reported xanthochromia in 30% of cases, frequently without associated bleeding. In those patients, xanthochromia correlated with high-density lesions on imaging, suggesting a link to hemorrhagic necrosis or elevated CSF protein [7].

In this case, a CSF red blood cell count of zero and normal cranial imaging effectively ruled out a hemorrhagic process. Xanthochromia is therefore more likely attributed to raised CSF protein levels, although these were marginally below the threshold commonly associated with Froin’s syndrome. Notably, CSF protein is typically only mildly elevated in viral encephalitis, and the level in this case (139.5 mg/dL) exceeds what is typically observed in infection alone [10].

Yamahata et al. reported that patients with lumbar spinal stenosis and medium block to contrast had significantly elevated CSF protein levels (104.3 ± 59 mg/dL) compared to those without obstruction [12]. Our patient had chronic cervical spinal stenosis and cervicomedullary narrowing, which may have similarly impaired CSF circulation and contributed to protein elevation. Furthermore, Froin's syndrome with impaired CSF circulation at the cervical level has been reported previously [7].

Given the absence of other inflammatory markers-such as pleocytosis, fever, or altered mental status, the elevated protein level is best explained by a combination of impaired CSF flow due to mechanical obstruction and a mild viral component. We therefore propose that the xanthochromia in this case is likely due to a mixed etiology involving both partial CSF obstruction and subclinical VZV infection.

Limitations

This case report has several limitations. A repeat lumbar puncture was not performed, which could have clarified whether the CSF abnormalities were transient or persistent. Furthermore, although VZV DNA was detected in the CSF, the absence of definitive clinical or radiological features of encephalitis limits interpretation of its pathogenic role. Additional viral markers, such as intrathecal antibody production (IgG/IgM), would have enhanced diagnostic certainty.

## Conclusions

This case highlights the diagnostic complexity of cerebrospinal fluid (CSF) xanthochromia in the absence of subarachnoid hemorrhage. While xanthochromia traditionally signals bleeding, this patient’s normal imaging, absence of red blood cells, and modestly elevated CSF protein pointed away from hemorrhagic causes. The detection of varicella zoster virus (VZV) in the CSF - despite the lack of overt encephalitic features - raises the possibility of a subclinical viral infection as the primary contributor. However, coexisting cervicomedullary narrowing and a history of inflammatory disease suggest that impaired CSF dynamics and chronic inflammation may have also played a role. Ultimately, this case underscores the importance of considering a broad differential for xanthochromia, including infectious, mechanical, and inflammatory etiologies, particularly when classic findings of SAH are absent. 
